# Histopathological Confirmed Polycythemia Vera with Transformation to Myelofibrosis Depicted on [^18^F]FDG PET/CT

**DOI:** 10.3390/diagnostics14100982

**Published:** 2024-05-08

**Authors:** Moritz B. Bastian, Arne Blickle, Caroline Burgard, Octavian Fleser, Konstantinos Christofyllakis, Samer Ezziddin, Florian Rosar

**Affiliations:** 1 Department of Nuclear Medicine, Saarland University, 66421 Homburg, Germany; arne.blickle@uni-saarland.de (A.B.); caroline.burgard@uks.eu (C.B.); samer.ezziddin@uks.eu (S.E.); florian.rosar@uks.eu (F.R.); 2 Department of Hematology, Oncology, Clinical Immunology, Rheumatology, Saarland University, 66421 Homburg, Germany; octavian.fleser@uks.eu (O.F.); konstantinos.christofyllakis@uks.eu (K.C.)

**Keywords:** polycythemia vera, myelofibrosis, FDG, PET/CT

## Abstract

We present a case of a 59-year-old male diagnosed with polycythemia vera (PV) for many years, who presented with a relatively abrupt onset of heavy constitutional symptoms, including fatigue, night sweats, and a 10% weight loss over 6 weeks. Despite the known initial diagnosis of PV, the presence of profound B-symptoms prompted further investigation. A positron emission tomography/computed tomography (PET/CT) scan with ^18^F-Fluorodeoxyglucose ([^18^F]FDG) was performed to exclude malignant diseases. The [^18^F]FDG PET/CT revealed intense metabolic activity in the bone marrow of the proximal extremities and trunk skeleton, as well as a massively enlarged spleen with increased metabolic activity. Histopathologically, a transformation to myelofibrosis was revealed on a bone marrow biopsy. The case intends to serve as an exemplification for [^18^F]FDG PET/CT in PV with transformation to myelofibrosis (post-PV myelofibrosis).

**Figure 1:**
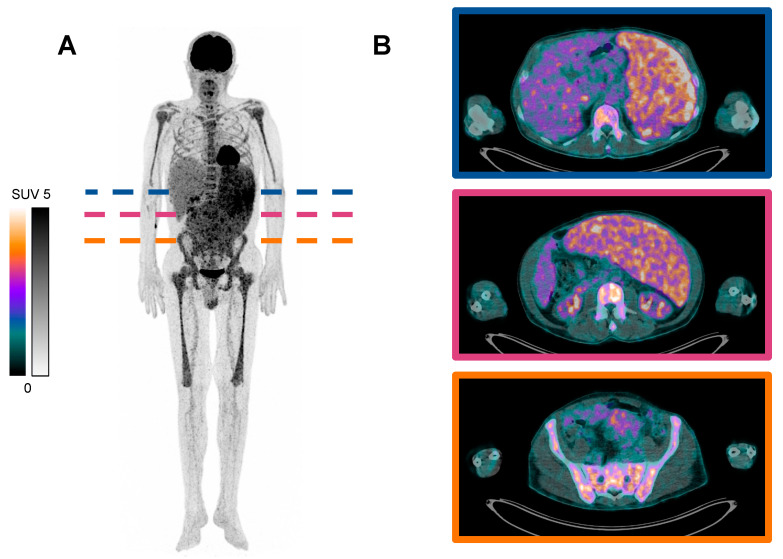
^18^F-Fluorodeoxyglucose ([^18^F]FDG) targeted positron emission tomography/computed tomography (PET/CT) scan of a patient with advanced polycythemia vera (PV) and transformation to myelofibrosis. A 59-year-old male with a known history of PV presented with newly developed profound constitutional symptoms, including fatigue, night sweats, and a 10% weight loss over 6 weeks. Despite the known initial diagnosis of PV, the abrupt deterioration of his general condition and new onset of B-symptoms prompted further investigation, and the patient was referred to the nuclear medicine department for further diagnostics regarding malignancy. Subsequently, a [^18^F]FDG PET/CT (acquired 65 min post-injection of 225 MBq) was performed: (**A**) maximum intensity projection (MIP), and (**B**) three transversal slices of [^18^F]FDG PET/CT, showing intense metabolic activity in the bone marrow of the proximal extremities and trunk skeleton, as well as an enlarged spleen with increased metabolic activity. (**B**) shows the specific extent of splenomegaly from the upper abdominal to the pelvic region with increased metabolic activity. No evidence of a secondary malignancy was found. Bone marrow biopsy was conducted, and histopathological examination revealed hypercellularity with increased erythroid, myeloid, and thromboid lineages with grade 2 fibrosis (MF-2 according to European consensus grading [1]). Additional molecular studies detected *JAK2* V617F mutation. Thereby, the diagnosis of advanced PV with transformation to post-PV myelofibrosis was determined. Polycythemia vera (PV) is a clonal myeloproliferative neoplasm (MPN) characterized by erythrocytosis, often accompanied by leukocytosis and/or thrombocytosis. It belongs to the group of chronic myeloproliferative neoplasms (MPN), according to WHO/ICC 2022 [2]. Genetic alterations of the hematopoietic stem cell are the underlying pathomechanism, causing a clonal proliferation of one or more terminally differentiated cell lineages in the peripheral blood. Major diagnostic criteria for the diagnosis of PV include elevated hemoglobin concentration and/or hematocrit, the presence of *JAK2* V617F or *JAK2* exon 12 mutations, and trilineage hyperplasia (panmyelosis) on a bone marrow biopsy [3]. PV can evolve into myelofibrosis (post-PV myelofibrosis) or acute myeloid leukemia (AML) [4]. Approximately 50% of patients will progress to post-PV myelofibrosis 20 years after disease onset. In some cases, patients may present with profound B-symptoms, leading to concerns about an independent second underlying malignancy. In this scenario [^18^F], FDG PET/CT imaging has a high differential diagnostic value by identifying metabolic abnormalities in tissue as malignancy [5,6] or, as in this case, aiding in the detection of the shift to spent-phase PV with subsequent myelofibrosis [7]. The potential of [^18^F]FDG in the management of PV was only reported for a limited number of patients [8,9,10] and is not established as a standard imaging modality. Depending on the severity of PV, different levels of uptake in the bone marrow and spleen are reported and may be characteristic [10,11,12,13]. The heightened metabolic activity PV is primarily attributed to the increased cellular turnover and proliferation within the bone marrow, as well as the enlarged spleen, which is actively involved in hematopoiesis and sequestration of blood cells. The case intends to serve as an exemplification for [^18^F]FDG PET/CT in PV with transformation to myelofibrosis (post-PV myelofibrosis). Future studies, ideally in prospective settings, should be performed evaluating the value of [^18^F]FDG PET/CT in clinical practice, i.e., diagnostic and treatment of PV.

## Data Availability

The datasets, including the DICOM image file used and analyzed in this paper, are available from the corresponding author upon reasonable request.
